# Early predictors of visual and axonal outcomes after acute optic neuritis

**DOI:** 10.3389/fneur.2022.945034

**Published:** 2022-09-08

**Authors:** Minh N. L. Nguyen, Chao Zhu, Scott C. Kolbe, Helmut Butzkueven, Owen B. White, Joanne Fielding, Trevor J. Kilpatrick, Gary F. Egan, Alexander Klistorner, Anneke van der Walt

**Affiliations:** ^1^Department of Neurosciences, Monash University, Melbourne, VIC, Australia; ^2^Department of Neurology, Alfred Health, Melbourne, VIC, Australia; ^3^Department of Neurology, Royal Melbourne Hospital, Melbourne, VIC, Australia; ^4^Monash Biomedical Imaging, Monash University, Melbourne, VIC, Australia; ^5^Save Sight Institute, University of Sydney, Sydney, NSW, Australia

**Keywords:** optic neuritis, prognosis, OCT, MRI, Ishihara

## Abstract

**Background:**

Predicting long-term visual outcomes and axonal loss following acute optic neuritis (ON) is critical for choosing treatment. Predictive models including all clinical and paraclinical measures of optic nerve dysfunction following ON are lacking.

**Objectives:**

Using a prospective study method, to identify 1 and 3 months predictors of 6 and 12 months visual outcome (low contrast letter acuity 2.5%) and axonal loss [retinal nerve fiber layer thickness and multifocal evoked potential (mfVEP) amplitude] following acute ON.

**Methods:**

In total, 37 patients of acute ON onset were evaluated within 14 days using between-eye asymmetry of visual acuity, color vision (Ishihara plates), optical coherence tomography, mfVEP, and optic nerve magnetic resonance imaging [magnetic transfer ratio (MTR) and diffusion tensor imaging (DTI)].

**Results:**

Visual outcome at 6 and 12 months was best predicted by Ishihara asymmetry at 1 and 3 months following ON onset. Axonal loss at 6 and 12 months was reliably predicted by Ishihara asymmetry at 1 month. Optic nerve MTR and DTI at 3 months post-acute ON could predict axonal loss at 6 and 12 months.

**Conclusions:**

Simple Ishihara asymmetry testing 1 month after acute ON onset can best predict visual outcome and axonal loss at 6 and 12 months in a clinical or research setting.

## Introduction

Acute optic neuritis (ON) is a condition caused by inflammation of the optic nerve, leading to loss of visual acuity, color vision, peripheral field loss, and painful eye movements. Although recovery is generally good (1), many patients have long-term residual deficits which can impact patient quality of life, namely, reduced visual acuity (VA), contrast sensitivity, color perception, and visual field defects (2). There is still an unmet need for effective ON treatment to prevent permanent retinal ganglion cell (RGC) loss from axonal degeneration and demyelination (3). Predicting patients who will have poor recovery in the long term is of utmost importance in selecting patients early in their disease course who will benefit from these treatment trials. Due to its close association with multiple sclerosis (MS), intervention after acute and/or chronic ON is now an established clinical trial model for phase studies of potential neuroprotective and remyelinating treatments (4–8), However, the current literature is conflicting and incomplete for choosing a reliable predictor.

At present, the most sensitive clinical outcome measures used in assessing visual dysfunction are Sloan low contrast letter acuity (LCLA) and contrast sensitivity (CS), even when high contrast visual acuity (HCVA) appears to be normal (3, 9). Dysfunction of these two measures is associated with a reduction in retinal nerve fiber layer (RNFL) thickness, a surrogate marker for optic nerve axonal degeneration (10). Paraclinical tests to assess optic nerve structure (RNFL and GCL thinning) and function (myelination status) include optical coherence tomography (OCT) (11) and visual-evoked potential (VEP) amplitude, and latency (12), respectively. In the acute setting, especially the first 3 months, the RNFL measurement and VEP measures may be inaccurate due to inflammatory swelling and conduction block and therefore may not be useful as early predictors of long-term visual outcomes (13). Spectral-domain OCT, which can measure ganglion cell and inner plexiform layer thickness, can detect early thinning within weeks of ON and is a better measure of acute axonal loss (4, 14).

Advanced quantitative optic nerve magnetic resonance imaging (MRI) with the potential to predict long-term clinical outcomes of ON includes magnetization transfer ratio (MTR) (15) and diffusion tensor imaging (DTI) (16). The MTR represents the exchange between free protons in the tissue and those associated with macromolecules in myelin or axonal membranes (17). A reduction in the MTR has been shown to correlate with both myelin and axonal loss in histopathological studies (18, 19). Previous studies have described flux in MTR that could indicate optic nerve remyelination (15).

The DTI is an MRI method sensitive to microstructural changes in white matter that is associated with axonal and myelin degradation at baseline (within 2 weeks of ON presentation) (20). The DTI axial diffusivity was found to correlate with RNFL thinning and mfVEP loss of amplitude at 6 and 12 months, and VA recovery at 6 months (16). This suggests that interventions targeting the normalization of axial diffusivity early after ON may improve axonal outcomes.

Advanced MRI techniques have the potential to be used early in the course of ON as predictors of visual outcomes. To date, the performance of these MRI techniques in models that include comprehensive clinical visual assessments (color vision and visual acuity), OCT, and VEP is unknown. In this study, we comprehensively assessed clinical assessment, OCT, VEP, optic nerve MTR, and DTI to independently predict visual and axonal outcomes at 6 and 12 months after acute ON.

## Materials and methods

### Standard protocol approvals, registrations, and patient consents

This study was conducted in accordance with the Declaration of Helsinki and was approved by the Human Research Ethics Committee of the Royal Victorian Eye and Ear Hospital (recruitment and clinical testing site) and Melbourne Health (clinical management site) and the Murdoch Children's Research Institute (MRI scanning site). All the study participants provided voluntary, written consent.

### Participants

Over 2 years, this study recruited 40 adult participants from a tertiary ophthalmology hospital who presented within 2 weeks with their first episode of unilateral acute ON. Patients were studied at baseline (within 48 hours of presentation and before steroid administration), and at one, six, and 12 months. All the patients had a baseline MRI brain scan with at least two T2 hyperintense lesions indicating a high risk of subsequent development of MS. Patients with early relapsing-remitting MS (onset within 2 years) according to MRI-based criteria were included (21). Patients were excluded if they had other ophthalmological or neurological diseases and if they had further episodes of ON during the course of the study. As anti-aquaporin 4 (anti-AQP4) antibody or anti-myelin oligodendrocyte antibody (anti-MOG Ab) were not freely available at the start of the study, we excluded patients with atypical ON and those without at least two demyelinating lesions on MRI to limit the inclusion of patients with NMOSD and MOG-antibody disease. A total of 37 patients remained after two patients elected to withdraw after 1 month and one patient was excluded after developing recurrent ON.

### Clinical assessment

The best corrected visual acuity was performed in optimal lighting conditions in the same room at each visit using high contrast (Sloan 100%) visual acuity (HCVA) and low contrast (Sloan 2.5%) letter acuity (LCLA) charts (22, 23). LogMAR scale equivalent scores were derived from the HCVA scores for interpretation. In the LCLA tests, the numbers of letters correctly recognized (maximum 60/chart) were recorded for each eye. Finally, 38 Ishihara plates were used to assess color vision of each eye.

### Optical coherence tomography

At the time of this study, a time domain OCT (TD–OCT) scanner (Stratus™, software version 3.0, Carl Zeiss Meditec Inc., Dublin, CA) was used to measure RNFL thickness. This version was the only one available at the study center. The RNFL protocol consisted of three circular scans centered on the optic disc with 3.4 mm diameters. For quality control, the signal strength of seven or more was accepted.

### Multi-focal visual-evoked potential

All the mfVEPs were recorded using the Accumap™ (ObjectVision, Sydney, Australia) based on procedures described by Fraser et al. (24). The VEPs were calculated for each sector of the visual field and for the whole eye using the OPERA program (ObjectVision, Sydney, Australia). We excluded segments with an amplitude signal less than 1.96 times the standard deviation (SD) of the trace within the interval 400–1,000 ms as they were considered as non-recordable.

### Magnetic resonance imaging

The MRI was performed on a Siemens 3T Trio MRI system with a 32-channel transmit-receive head coil. The MTR of optic nerves was calculated from two coronal scans (2D gradient echo) using methods described in Wang et al. (15). Optic nerve DTI was calculated from a previously described protocol (25). The DTI values used for the analysis of axial and radial diffusivity of the affected and unaffected eye were averaged from 6 to 8 repeated measurements. The means for each patient were then used to calculate the overall mean for the patient population.

### Statistical analysis

Baseline characteristics and the clinical visual measures are reported as counts for discrete variables and mean (standard deviation [SD]) for continuous variables. To minimize the effect of inter-subject variability, we used the between-eye asymmetry of RNFL thickness, amplitude, and latency of the mfVEPs, LCLA 2.5%, MTR, and Ishihara as the study outcomes, which were calculated using the formula: (affected eye unaffected eye)/unaffected eye × 100. In the HCVA analysis, we used the values of the affected eye instead of between-eye asymmetry as the outcome since many participants had normal HCVA in the unaffected eye.

Univariate linear regression models were used to identify potential predictors of RNFL thickness, the amplitude and latency of mfVEPs, and LCLA 2.5% at 6 and 12 months. In total, five independent variables, namely, DTI axial and radial diffusivity, logMAR visual acuity, Ishihara, and MTR (1 and 3 months after onset) were used as predictive variables in the model, respectively. The variables with a *p*-value of < 0.1 in the univariate model were considered for inclusion in the multivariable analysis. The final multivariable models were constructed using a backward-stepwise approach. Age, sex, and corticosteroid treatment were also included in the final model. Baseline mfVEP amplitude asymmetry was adjusted in the multivariable model as it was significantly associated with mfVEP amplitude asymmetry at 6 and 12 months. We tested the normality of data using the Kolmogorov–Smirnov test, and all variables were normally distributed. The variance inflation factor (VIF) was calculated to detect multicollinearity.

In sensitivity analyses, the final model was re-run with missing data replaced using multiple imputations based on chained equations (Supplementary Table S1). In total, 20 imputed data sets were generated by using a sequential regression method. In all subsequent analyses, effect estimates were computed separately for each of the 20 data sets and then combined using the Rubin's rules.

All *p*-values were two-sided, and a *p*-value of < 0.05 was considered statistically significant. Analyses were performed with R, version 4.0.3 (R Foundation for Statistical Computing).

## Results

### Demographics and longitudinal measurements

The demographics and longitudinal measurements are presented in Table 1. The mean age of the patients was 35.76 years (± 8.99) with twice as many female participants as males. Most patients (75.68%) were treated with steroids acutely after the initial baseline assessment. In total, 42% of the patients converted to clinically definite MS at 12 months. At baseline, compared with the unaffected eye, the affected eye had worse HVCA, LCLA 2.5%, and color vision, and also elevated RNFL and reduced mfVEP amplitude. The MTR values at baseline for both eyes were similar.

**Table 1:** Demographic and baseline clinical visual measures of ON patients.

	**Total (*n*** = 37)****	**Affected eye**	**Unaffected eye**
Age (years)*	35.76 ± 8.99	-	-
**Sex (Male/Female)**	11/26	-	-
**Corticosteroid (N, %)**	28 (76)	-	-
**Days since symptom onset**	5.81 ± 3.62	-	-
**Conversion to clinically definite MS by 12 months (N, %)**	13 (42)		
**Visual parameters**			
**High contrast visual acuity (logMAR)**			
Baseline		0.63 ± 0.35	−0.13 ± 0.07
1 month		0.07 ± 0.27	−0.14 ± 0.07
3 month		−0.01 ± 0.22	−0.12 ± 0.08
6 month		−0.04 ± 0.21	−0.12 ± 0.08
12 month		−0.05 ± 0.18	−0.11 ± 0.10
**Low contrast visual acuity 2.5% (out of 60)**			
Baseline		3.84 ± 9.25	32.96 ± 8.40
1 month		17.81 ± 13.58	34.58 ± 6.95
3 month		21.19 ± 13.38	34.43 ± 7.15
6 month		25.71 ± 13.15	35.95 ± 7.28
12 month		27.31 ± 14.59	35.08 ± 8.46
**Ishihara (out of 38 plates)** *			
Baseline		13.08 ± 14.16	37.97 ± 0.16
1 month		27.44 ± 13.54	37.89 ± 0.46
3 month		31.73 ± 10.34	37.89 ± 0.39
6 month		34.46 ± 9.02	37.91 ± 0.37
12 month		35.70 ± 7.39	37.97 ± 0.16
**Average RNFL thickness (μm)**			
Baseline		130.62 ± 41.41	105.04 ± 17.81
1 month		110.8 ± 26.18	103.28 ± 11.64
3 month		91.92 ± 16.75	102.95 ± 13.91
6 month		86.29 ± 16.56	103.66 ± 13.63
12 month		84.22 ± 16.32	102.78 ± 13.65
**mfVEP amplitude (v)**			
Baseline		82.53 ± 46.66	162.65 ± 45.77
1 month		114.61 ± 53.64	184.57 ± 35.97
3 month		123.52 ± 51.16	170.54 ± 35.33
6 month		125.23 ± 45.73	171.46 ± 43.62
12 month		130.54 ± 43.94	173.68 ± 43.46
**MRI parameters**			
**Number of baseline MRI brain lesions**	7.89 ± 9.05	-	-
DTI (Axial)*			
Baseline		1.59 ± 0.26	1.80 ± 0.19
1 month		1.65 ± 0.21	1.81 ± 0.15
3 month		1.75 ± 0.24	1.84 ± 0.22
6 month		1.78 ± 0.15	1.78 ± 0.17
12 month		1.82 ± 0.17	1.74 ± 0.19
DTI (Radial)*			
Baseline		0.88 ± 0.16	0.92 ± 0.20
1 month		0.91 ± 0.17	0.93 ± 0.15
3 month		1.00 ± 0.19	0.94 ± 0.20
6 month		1.00 ± 0.15	0.92 ± 0.18
12 month		1.06 ± 0.17	0.91 ± 0.19
MTR*			
Baseline		43.17 ± 7.04	43.22 ± 6.83
1 month		42.79 ± 7.13	44.82 ± 7.57
3 month		41.17 ± 9.77	44.94 ± 7.44
6 month		37.71 ± 7.77	43.27 ± 6.32
12 month		38.74 ± 7.24	42.51 ± 7.20

The mean and standard deviation (Mean ± SD) * are shown if the variable is continuous. In contrast, the number of observations for each level is displayed if the variable is binary. SD, standard deviation; ON, optic neuritis; MS, Multiple sclerosis.

An analysis using baseline measures (at 0 months) was performed but there were no significant associations with 6-and 12-month outcome measures found. The following sections describe the associations with visual and axonal outcomes at 6 and 12 months using variables measured at 1 and 3 months.

### Predictors of visual outcome in ON (LCLA 2.5%)

The final multivariate model in Table 2 demonstrated that the best predictors of LCLA 2.5% asymmetry at 6 months were Ishihara and HCVA asymmetry at 1 and 3 months, whereas the best predictors of LCLA 2.5% asymmetry at 12 months were HCVA asymmetry at 1 month and the Ishihara asymmetry at 3 months. Note that only the relevant variables in the final models are included in this table, and subsequent tables.

**Table 2:** LCLA 2.5%^*^ asymmetry.

	**Univariable models**		**Multivariable models**			
	**Coefficient (SE)**, ***p*** **value**		**Coefficient (SE), *p* value**	**VIF^†^**	**Coefficient (SE), *p* value**	**VIF**
	**6-month**	**12-month**	**6-month**		**12-month**	
**1-month**						
MTR	0.14 (0.28), 0.62	0.15 (0.36), 0.68	-	-	-	-
DTI (Radial)	0.21 (0.41), 0.61	0.26 (0.53), 0.63	-	-	-	-
Ishihara	**0.62 (0.12)**, ** <0.01**	**0.74 (0.16)**, ** <0.01**	**0.36 (0.14), 0.01**	2.11	0.39 (0.21), 0.07	2.09
DTI (Axial)	0.75 (0.48), 0.13	0.44 (0.63), 0.50	-	-	-	-
HCVA (logMAR)	**−95.18 (13.22)**, ** <0.01**	**−112.86 (19.73)**, ** <0.01**	**−67.67 (16.89)**, ** <0.01**	1.86	**−80.47 (26.61), 0.01**	1.82
**3-month**						
MTR	**0.67 (0.31), 0.04**	0.69 (0.41), 0.10	-	-	-	-
DTI (Radial)	−0.27 (0.36), 0.47	−0.70 (0.44), 0.12	-	-	-	-
Ishihara	**0.82 (0.17)**, ** <0.01**	**1.05 (0.19)**, ** <0.01**	**0.46 (0.19), 0.02**	1.71	**0.76 (0.25)**, ** <0.01**	1.66
DTI (Axial)	0.84 (0.61), 0.18	0.21 (0.76), 0.79	-	-	-	-
HCVA (logMAR)	**−104.56 (18.89)**, ** <0.01**	**−112.03 (26.08)**, ** <0.01**	**−77.22 (23.67)**, ** <0.01**	1.88	−59.93 (31.66), 0.07	1.78

* Low contrast letter acuity 2.5%.

† Variance inflation factor.

Age, sex, and corticosteroid are adjusted in the multivariable models as confounders. Those factors with *p*-values less than 0.1 (*p*-value < 0.1) in the univariable models are included in the multivariable models. The backward stepwise approach is applied in the model selection.

### Predictors of axonal loss (RNFL and mfVEP amplitude) in ON

#### RNFL

The final multivariate model in Table 3 illustrated that the most reliable predictors of RNFL thickness asymmetry at both 6 and 12 months were Ishihara asymmetry at 1 month and MTR and axial diffusivity asymmetry at 3 months. Axial diffusivity asymmetry at 1 month could predict 12-month RNFL and radial diffusivity asymmetry at 3 months could predict RNFL asymmetry at 6 months only.

**Table 3:** Predictors of 6 and 12-month RNFL^*^ thickness asymmetry.

	**Univariable models**		**Multivariable models**			
	**Coefficient (SE)**, ***p*** **value**		**Coefficient (SE), *p* value**	**VIF^†^**	**Coefficient (SE), *p* value**	**VIF**
	**6-month**	**12-month**	**6-month**		**12-month**	
**1-month**						
MTR	−0.08 (0.12), 0.49	−0.02 (0.11), 0.89	-	-	-	-
DTI (Radial)	−0.04 (0.17), 0.81	0.03 (0.16), 0.88	-	-	-	-
Ishihara	**0.23 (0.05)**, ** <0.01**	**0.20 (0.05)**, ** <0.01**	**0.20 (0.06)**, ** <0.01**	1.18	**0.18 (0.06)**, ** <0.01**	1.19
DTI (Axial)	**0.38 (0.19), 0.05**	**0.45 (0.18), 0.02**	0.25 (0.17), 0.15	1.13	**0.37 (0.17), 0.04**	1.14
HCVA (logMAR)	**−22.60 (7.59)**, ** <0.01**	**−20.68 (7.71), 0.01**	-	-	-	-
**3-month**						
MTR	**0.30 (0.13), 0.03**	**0.35 (0.12)**, ** <0.01**	**0.27 (0.11), 0.02**	1.10	**0.34 (0.11)**, ** <0.01**	1.12
DTI (Radial)	−0.24 (0.13), 0.07	−0.15 (0.13), 0.26	**−0.35 (0.13), 0.01**	1.30	−0.21 (0.13), 0.12	1.31
Ishihara	**0.27 (0.07)**, ** <0.01**	**0.25 (0.07)**, ** <0.01**	**−**	**−**	**−**	**−**
DTI (Axial)	0.39 (0.23), 0.10	**0.45 (0.21), 0.04**	**0.60 (0.21)**, ** <0.01**	1.35	**0.64 (0.21)**, ** <0.01**	1.35
HCVA (logMAR)	**−23.70 (8.99), 0.01**	**−22.02 (9.14), 0.02**	**−**	**−**	**−**	**−**

* Retinal nerve fibre layer.

† Variance inflation factor.

Age, sex, and corticosteroid are adjusted in the multivariable models as confounders. Those factors with *p*-values less than 0.1 (*p*-value < 0.1) in the univariable models are included in the multivariable models. The backward stepwise approach is applied in the model selection.

#### mfVEP amplitude

In the final model shown in Table 4, Ishihara asymmetry at 1 and 3 months correlated with mfVEP asymmetry at 6 and 12 months. Axial diffusivity asymmetry at 1 month could predict mfVEP asymmetry at 12 months. The MTR asymmetry at 3 months was able to predict mfVEP asymmetry at 6 months. HVCA asymmetry at 1 and 3 months did not show a significant correlation with mfVEP asymmetry in the multivariate analysis.

**Table 4:** mfVEP^*^ amplitude asymmetry.

	**Univariable models**		**Multivariable models**			
	**Coefficient (SE)**, ***p*** **value**		**Coefficient (SE), *p* value**	**VIF^†^**	**Coefficient (SE), *p* value**	**VIF**
	**6-month**	**12-month**	**6-month**		**12-month**	
**1-month** ^**‡**^						
MTR	−0.14 (0.24), 0.55	−0.08 (0.21), 0.71	-	-	-	-
DTI (Radial)	0.14 (0.31), 0.66	0.20 (0.29), 0.50	-	-	-	-
Ishihara	**0.77 (0.26), 0.01**	**0.79 (0.27), 0.01**	**0.27 (0.10), 0.01**	1.18	**0.30 (0.09)**, ** <0.01**	1.17
DTI (Axial)	**0.85 (0.34), 0.02**	**0.98 (0.30)**, ** <0.01**	0.44 (0.31), 0.16	1.12	**0.60 (0.27)**, ** <0.01**	1.16
HCVA (logMAR)	**−42.9 (14.42), 0.01**	**−40.47 (13.04)**, ** <0.01**	-	-	-	-
**3-month** ^**‡**^						
MTR	0.44 (0.26), 0.10	0.39 (0.23), 0.10	**0.54 (0.20), 0.02**	1.30	0.40 (0.21), 0.07	1.38
DTI (Radial)	−0.45 (0.25), 0.08	−0.33 (0.25), 0.20	-	-	-	-
Ishihara	**0.55 (0.12)**, ** <0.01**	**0.58 (0.11)**, ** <0.01**	**0.27 (0.12), 0.04**	1.36	**0.34 (0.14), 0.02**	1.44
DTI (Axial)	0.43 (0.43), 0.32	0.50 (0.38), 0.21	-	-	-	-
HCVA (logMAR)	**−53.05 (16.00)**, ** <0.01**	**−49.71 (14.91)**, ** <0.01**	-	-	-	-

* Multifocal Visual Evoked Potential.

† Variance inflation factor.

‡ Baseline mfVEP amplitude asymmetry is adjusted in the final model as it is significantly associated with mfVEP amplitude asymmetry at 6 and 12 months.

Age, sex, and corticosteroid are adjusted in the multivariable models as confounders. Those factors with *p*-values less than 0.1 (*p*-value < 0.1) in the univariable models are included in the multivariable models. The backward stepwise approach is applied in the model selection.

## Discussion

This prospective study used multivariable models to assess the performance of clinical and paraclinical tests as early predictors of ON-associated visual and axonal loss. The visual outcome measured as low contrast letter acuity (LCLA 2.5%) at both 6 and 12 months, was best predicted by reduced color vision (Ishihara test) and high contrast visual acuity at 1 and 3 months after onset. Axonal loss, measured by RNFL and mfVEP amplitude at 6 and 12 months, was also reliably predicted by decreased color vision at 1 month after onset of ON. In total, 3 months after acute ON, advanced MRI modalities (optic nerve MTR and DTI axial diffusivity), predicted RNFL thickness asymmetry at 6 and 12 months. The mfVEP asymmetry could also be predicted at 6 and 12 months, respectively, by the MTR asymmetry at 3 months and axial diffusivity at 1 month post ON onset. The findings of this study are consistent with our previous study, in which we defined optic nerve MTR asymmetry at 3 months as a predictor of axonal loss (15).

Acute ON remains a common phase II clinical trial model for neuroprotective and/or remyelinating therapies in patients at risk of, or with MS (4–8). These clinical trials studied varying primary and secondary outcomes, concentrating on either visual loss, or axonal loss, but have not used early assessments as a selection strategy to identify participants who would have poorer outcomes after acute ON. Enriching these challenging studies with participants who are most likely to benefit from early intervention is critical to demonstrate the true effect of a trial intervention to reduce the proportion of patients in a trial who will improve spontaneously.

This study demonstrates that color vision 1 month from the onset of ON is an excellent predictor of both visual outcome and optic nerve axonal loss at 6 and 12 months. During the course of typical ON, mixed spectral dyschromatopsia occurs early with blue-green defects being slightly more common in the acute phase, and red-green defects more common at 6 months (26). Previous studies have not assessed color vision deficits as a predictor of visual loss. In this study, color vision at the onset of ON correlates with poorer visual and axonal outcomes up to 12 months. Using Ishihara plates to test color vision is simple and requires no additional training, making it an attractive clinical measure to guide clinical management. In an ideal setting, early intervention in acute ON is favorable as most loss of GCL occurs within 1 month (27) and a delay in potential therapies may lead to reduced efficacy. However, stratification tools at 1-month are still valuable in identifying patients eligible for remyelination therapies and are often more pragmatic. Our study suggests clinical measurement of color vision at 1-month help predict 6- and 12 month ON outcomes as we did not find any associations at baseline. Impaired color vision at 1 month after ON may also be an easily executable stratification tool to identify participants early in the course of ON trials, particularly remyelinating trials for patients who have undergone early axonal loss resulting from acute ON.

We also recognize some limitations with Ishihara testing. Although each Ishihara plate has high sensitivity and specificity (between 0.85– 0.95) for red–green color-blindness, the test is less sensitive for detecting mild deuteranopia and is unable to detect tritanopia (28), both of which can occur in patients with ON. The sensitivity and specificity of Ishihara plates could be enhanced by adjunctive use of the Hardy-Rand-Rittler (HRR) pseudoisochromatic test (29). Both HRR and Ishihara tests are affected by decreased contrast sensitivities, and therefore may falsely identify color deficiencies in patients who otherwise only have decreased contrast sensitivity. However, the Ishihara test performs better with a lower percentage of errors in patients with decreased contrast sensitivity than both the HRR and the Farnsworth D-15 test used alone, due to larger numbers of the Ishihara test plates (30). Another potential limitation of Ishihara testing is a floor effect where patients on a spectrum of severity of color loss can score similarly with no correct plates read. Thus, the actual 6- and 12 month visual and axonal outcomes in these cases may vary despite all having the same predicted outcomes early on.

In addition to Ishihara testing, this study found HCVA at 1 and 3 months after acute ON can be used to predict long-term low-contrast acuity visual outcome, used as a primary or secondary outcome measure in several phase II trials (4, 7, 8). Although HCVA can be easily measured in early patient recruitment, it is limited by a ceiling effect and thus, not commonly used as an outcome measure in isolation (31).

This study demonstrated that the MTR asymmetry and axial diffusivity asymmetry at 3 months best-predicted axonal loss in the form of RNFL asymmetry at 6 and 12 months, and mfVEP amplitude at 6 months. Thus, studies looking at RNFL as an axonal outcome, rather than mfVEP, may benefit more from early imaging studies to predict 12-month outcomes. Recruitment of acute patients with ON into phases II and III trials is challenging because of a large number of early screening and baseline tests that generally must be completed in a short amount of time. It is therefore not surprising that chronic ON models are also being used such as in VISIONARY-MS (32). Our results suggest that stratification to identify those at risk of worse visual and axonal outcomes, could be performed even 3 months after acute ON using optic nerve MTR or axial diffusivity asymmetry, therefore extending the window for trial stratification.

The MTR can be influenced by other pathological processes such as inflammation and edema in acute demyelination which may affect true MTR measurements (33). However, this influence has only been observed in whole brain MTR and in animal studies, not in isolated human optic nerve MTR. We observed that most patients in this study were on steroids from the onset of their acute ON and postulate that MTR measurements may be more informative at 3 months post onset of acute ON due to the reduction of inflammation and edema from steroid treatment. The whole brain MTR is also decreased in isolated ON, specifically in the visual cortex, possibly because of the trans-synaptic neuronal degeneration or cortical synaptic changes (34). The effects of intervention in ON trials on the whole brain MTR may be difficult to interpret as it can be influenced by general cortical atrophy and lesion load (34). The optic nerve MTR asymmetry appears to be less influenced by the post-chiasmal changes by minimizing inter-subject variability and allowing changes to the affected eye to be determined (15). Neither whole brain nor optic nerve MTR has been applied in ON trials. The current MRI outcome measures in ON neuroprotective trials include the number of gadolinium-enhancing lesions on MRI brain (4) or optic nerve diameter (35), thus, incorporation of advanced MRI techniques such as MTR may assist with interpreting treatment effect. The benefits of optic nerve MTR imaging include acquisition in a short time (within 8 min as it was done in this study) and minimal post-processing. It is also readily available to be done on most MRI systems.

Similar to our previous study using optic nerve DTI, this study found that greater axial diffusivity asymmetry of the optic nerve at 3 months was useful at predicting axonal loss at 6 and 12 months, but without an LCLA 2.5% correlation (16). There was, however, a trend between axial diffusivity asymmetry with persistent loss of HCVA at 6 and 12 months, but this is not as clinically useful compared to LCLA 2.5% as an outcome measure which is overall more sensitive to treatment effect (31). The optic nerve axial diffusivity at 3 months, like MTR, can therefore be a useful predictor of RNFL thinning at 6 and 12 months.

We acknowledge that this study has limitations regarding to small sample size. In addition, measurements of RNFL were performed on a TD–OCT which has several limitations compared to the more sensitive spectral domain OCT (SD–OCT). It has a lower spatial resolution of 10–15 μm, due to the absence of eye tracking and slower image acquisition speed, compared to the SD–OCT axial resolution of ~ 5 μm because of the faster image acquisition and eye tracking technology (36). The SD–OCT is preferable for use in future trials as it acquires images up to 50 times faster (36). Ganglion cell layer (GCL) thickness was not examined with OCT for this study, but we acknowledge that GCL may be a more sensitive marker of early axonal loss in ON compared with the RNFL and thus, we suggest further exploration with larger sample size and use of an SD–OCT (37).

Multifocal VEP amplitude, rather than latency was used as an outcome because it more strongly predicts final axonal loss and has a better correlation with inter-eye asymmetry values of RNFL thickness (38). We also studied a cohort of patients with a single episode or first episode of clinical demyelination at risk of developing RRMS or with early RRMS when applying the new McDonald criteria. Therefore, our results may not be applicable to patients with longstanding MS who develop ON. Despite careful clinical assessment of ON, we did not systematically test for anti-AQP-4 antibody and anti-MOG antibody as these tests were not freely available at the time. This is important to consider as NMOSD and MOG-antibody disease ON can overlap with typical MS-related ON but has a different temporal and clinical course that could have affected our results.

## Conclusion

This study demonstrates that simple Ishihara testing of color vision at 1-month post-acute ON can predict both visual and axonal outcomes 6 and 12 months, with advanced MRI measures at 3 months as an alternative. These methods may be used to guide clinical management and assist future researchers in selecting patients that would most benefit from neuroprotective and remyelination ON trials.

## Data availability statement

The raw data is accessible following approval by the relevant IRB. Requests to access the datasets should be directed to the corresponding author.

## Ethics statement

The studies involving human participants were reviewed and approved by the Human Research Ethics Committee of the Royal Victorian Eye and Ear Hospital (recruitment and clinical testing site) and Melbourne Health (clinical management site) and the Murdoch Children's Research Institute (MRI scanning site). The patients/participants provided their written informed consent to participate in this study.

## Author contributions

MN, CZ, SK, and AW were responsible for the collection of data, drafting and revision of the manuscript for content, including medical writing for content, analysis, and interpretation of data. HB, OW, JF, TK, GE, and AK were responsible for revision of the manuscript. All authors contributed to the article and approved the submitted version.

## Funding

This research was supported by the National Health and Medical Research Council of Australia (grant 628415).

## Conflict of interest

Author HB's institution (Monash University) received compensation for consulting, talks, and advisory/steering board activities from Alfred Health, Biogen, Genzyme, Merck, and Novartis research support from Biogen, Merck, MS Research Australia, National Health and Medical Research (Australia), Novartis, the Oxford Health Policy Forum, and Roche. He has received personal compensation for steering group activities from the Oxford Health Policy Forum and Merck. Author JF has received funding from Genzyme, Biogen, and Novartis, and received honoraria from Novartis. Author TK has served on scientific advisory boards for GlaxoSmithKline, Neurosciences Victoria, and the Victorian Neurotrauma Initiative has received funding for travel from Bayer Schering pharma, Sanofi Aventis, Merck Serono, and Novartis served on the editorial board of Therapeutic Advances in Neurological Disorders is listed as an inventor on patents re: HIV test kit method for detecting anti-HIV-1 antibodies in saliva a method of modulating cell survival and reagents useful for the same. Methods for the treatment and prophylaxis of demyelinating disease and method of treatment in the field of inflammatory neurodegeneration and receives research support from Bayer Schering Pharma, Biogen IDEC. Author GE is Deputy Chairperson of the International Neuroinformatics Coordinating Facility and a member of the Neurosciences Victoria Scientific Advisory Board. He has received travel expenses and/or honoraria for lectures or educational activities not funded by industry. He is Deputy Editor in Chief of the journal Human Brain Mapping, and he serves on the editorial board of Neuroinformatics. Author AW served on advisory boards and receives unrestricted research grants from Novartis, Biogen, Merck, and Roche. She has received speaker's honoraria and travel support from Novartis, Roche, and Merck. She receives grant support from the National Health and Medical Research Council of Australia and MS Research Australia. The remaining authors declare that the research was conducted in the absence of any commercial or financial relationships that could be construed as a potential conflict of interest.

## Publisher's note

All claims expressed in this article are solely those of the authors and do not necessarily represent those of their affiliated organizations, or those of the publisher, the editors and the reviewers. Any product that may be evaluated in this article, or claim that may be made by its manufacturer, is not guaranteed or endorsed by the publisher.
