# Supplementary material for: Early predictors of visual and axonal outcomes after acute optic neuritis
Source: Front Neurol. 2022 Sep 8;13:945034. doi: 10.3389/fneur.2022.945034 (PMC9493016; doi:10.3389/fneur.2022.945034)
Supplement: Supplementary file 1 [file Table_1.docx]

| Table S1  The associations of clinical visual measures at 1 and 3 months and primary outcomes (RNFL, mfVEP amplitude, and LCVA 2.5%) at 6 and 12 months after multiple imputations for missing data. | | | | | | |
| --- | --- | --- | --- | --- | --- | --- |
|  | RNFL | | mfVEP | | LCVA 2.5% | |
|  | Coefficient (SE), p value | | Coefficient (SE), p value | | Coefficient (SE), p value | |
|  | 6 month | 12 month | 6 month | 12 month | 6 month | 12 month |
| 1 month |  |  |  |  |  |  |
| MTR |  |  |  |  |  |  |
| DTI (Radial) |  |  |  |  |  |  |
| Ishihara | **0.19 (0.06), <0.01** | **0.18 (0.06), <0.01** | **0.27 (0.11), 0.03** | **0.28 (0.10), 0.01** | 0.34 (0.17), 0.06 | 0.39 (0.23), 0.10 |
| DTI (Axial) | 0.23 (0.19), 0.23 | **0.37 (0.17), 0.04** | 0.38 (0.34), 0.26 | **0.56 (0.29), 0.05** |  |  |
| Visual acuity (logMAR) |  |  |  |  | **-69.57 (20.57), <0.01** | **-79.90 (28.72), <0.01** |
|  |  |  |  |  |  |  |
| 3 month |  |  |  |  |  |  |
| MTR | **0.20 (0.13), 0.15** | **0.27 (0.11), 0.03** | 0.28 (0.22), 0.24 | 0.27 (0.18), 0.14 |  |  |
| DTI (Radial) | **-0.36 (0.14), 0.02** | **-0.29 (0.13), 0.04** |  |  |  |  |
| Ishihara |  |  | **0.38 (0.13), <0.01** | **0.36 (0.15), 0.02** | **0.48 (0.22), 0.04** | **0.75 (0.27), <0.01** |
| DTI (Axial) | **0.63 (0.24), 0.02** | **0.69 (0.22), <0.01** |  |  |  |  |
| Visual acuity (logMAR) |  |  |  |  | **-76.56 (27.50), 0.01** | -59.71 (33.85), 0.08 |
| RNFL: Retinal nerve fibre layer; mfVEP: Multifocal Visual Evoked Potential; LCVA 2.5%: Low contrast visual acuity 2.5%; MTR: Magnetization transfer ratio; DTI: Diffusion tensor imaging.  Age, sex, and corticosteroid are adjusted in the multivariable models as confounders. The between-eye asymmetries of RNFL, mfVEP, LCVA 2.5%, MTR, and Ishihara were used as outcomes in the fit of models. | | | | | | |
